# Automated deep learning based detection of cellular deposits on clinically used ECMO membrane lungs

**DOI:** 10.3389/fbinf.2026.1771574

**Published:** 2026-04-01

**Authors:** Daniel Pointner, Michael Kranz, Maria Stella Wagner, Moritz Haus, Karla Lehle, Lars Krenkel

**Affiliations:** 1 Department of Biofluid Mechanics, Faculty of Mechanical Engineering, Technical University of Applied Sciences (OTH) Regensburg, Regensburg, Germany; 2 Regensburg Center of Biomedical Engineering, Facility of University Regensburg, Technical University of Applied Sciences (OTH) Regensburg, Regensburg, Germany; 3 Department of Cardiothoracic Surgery, University Hospital Regensburg, Regensburg, Germany; 4 Department of Internal Medicine II, University Hospital Regensburg, Regensburg, Germany

**Keywords:** computer vision, deep learning, ECMO, histology, membrane lungs

## Abstract

**Introduction:**

Despite the promising application of extracorporeal membrane oxygenation (ECMO) in the treatment of critically ill patients, coagulation-associated technical complications, primarily clot formation and critical bleeding, remain a major challenge during ECMO therapy. The deposition of nucleated cells on the surface has been shown, yet the role of these cells towards complication development is still matter of ongoing research. In particular, the membrane lung (MemL) is prone to clot formation. Therefore, the investigation of nuclear deposits on its hollow-fibers may provide insights for a better understanding of the cellular mechanisms involved in the development of ECMO complications.

**Methods:**

To support current research, this study aimed to develop a deep learning–based tool for the automated detection and quantitative analysis of nuclear depositions on MemL hollow-fiber mats. A customized fluorescence microscopy workflow, combined with a semi-automated iterative labeling strategy, was used to generate a high-quality dataset for model training.

**Results:**

Six configurations of instance segmentation models were evaluated, with a Mask R-CNN with ResNet 101 backbone using dilated convolution providing the most balanced performance in both nuclei count and area accuracy. Compared with U-Net–based approaches such as Cellpose or StarDist, the proposed model demonstrated superior segmentation of overlapping and low-intensity nuclei, maintaining accuracy even in densely packed cellular regions.

**Discussion:**

We present an automated image analysis tool for clinically used MemLs, which exhibit complex three-dimensional hollow-fiber architectures and irregular cellular deposits that challenge conventional tools. A dedicated graphical user interface enables streamlined detection, morphometric analysis, and spatial clustering of nuclei, establishing a reproducible workflow for high-throughput analysis of fluorescence microscopy images. This approach eliminates labor-intensive manual counting and facilitates large-scale studies on cell-fiber interactions and disease-related correlations.

## 1 Introduction

Extracorporeal membrane oxygenation (ECMO) is a form of temporary mechanical circulatory or respiratory support used in patients with life-threatening cardiac and/or pulmonary failure. It is most often indicated in emergency or urgent settings as a last resort treatment option for critically ill adults, children, and neonates. ECMO utilizes a centrifugal pump to drain blood from the patient’s venous system which transports it to the membrane lung (MemL) for gas exchange. The MemL is comprised of numerous semi-permeable membrane hollow-fibers flushed with an oxygen-rich gas mixture. The resulting concentration gradient induces the gas exchange. Subsequently, the oxygenated blood is returned the patient either into the arterial or venous blood vessels depending on the required organ support. Veno-arterial ECMO (VA ECMO) provides circulatory support and is indicated in cardiogenic shock or during resuscitation at cardiac arrest (Bakhtiary et al., 2008). Alternatively, veno-venous ECMO (VV ECMO) focuses only on gas exchange of the blood and is a temporary treatment option for refractory respiratory failure like acute respiratory distress syndrome (Flörchinger et al., 2008).

Within the recent corona virus pandemic, ECMO has become more commonly known as a life-saving strategy for severe COVID-19 cases. Data from the Extracorporeal Life Support Organization (ELSO) Registry, the world’s largest ECMO patient database, demonstrates the rapid global expansion of ECMO use. The number of active ECMO centers and annual ECMO runs has steadily increased over the past decade. With the live ELSO dashboard reporting over 248,000 total ECMO cases submitted worldwide to date covering neonatal, pediatric, and adult patients across all support modes (Extracorporeal Life Support Organization (ELSO), 2025). This upward trend highlights ECMO’s growing clinical relevance and the increasing demand for optimized device performance and monitoring in a therapy that is increasingly used.

Despite its potentially significant beneficial effects on patients outcome and mortality (Peek et al., 2009; Sameed et al., 2019), it is highly invasive, resource-intensive, challenging for the clinical staff, and still associated the a variety of technical limitations. For example, severe coagulation-associated complications persist as a substantial limitation (Krivitski et al., 2018; Lubnow et al., 2014; Seeliger et al., 2020). Beyond the risk of severe bleeding, the progressive formation of clots in either the patient or within the ECMO circuit poses a major concern (Oliver, 2009; Murphy et al., 2015; Mulder et al., 2018; Lubnow et al., 2014). It is notable that the clotting complications occur even though patients are routinely managed with systemic anticoagulation (Seeliger et al., 2020) as well as with MemLs using anticoagulant heparin-coating, highlighting the complexity of blood–material interactions under extracorporeal circulation. Consequently, it is used only when recovery, transplantation, or transition to durable mechanical support is otherwise impossible.

In particular, every third of ECMO patient is requiring an exchange of the ECMO system, most of which (86 %) due to progressive or acute clot formation within the MemL, the essential part of the ECMO circuit, in which the oxygenation of the patient's blood occurs (Lubnow et al., 2014). During the corona virus pandemic, even higher levels of clot formation were observed (43% (Lehle et al., 2022), 64% (Bemtgen et al., 2021) in COVID patients with ECMO. Intra-device clot formation can directly impair device function and promote device-associated coagulation disorders, often necessitating urgent high-risk system exchange (Lehle et al., 2008; Lubnow et al., 2014; Frantzeskaki et al., 2023; Vajter and Volod, 2025). The MemL design gives rise to a complex blood flow situation, exerting an essential influence on coagulation. Elevated shear rates, for example, have been discussed to play a role in the process of clot formation in general (Springer, 2014; Kamada et al., 2017). At high shear rates, von Willebrand factor (vWF) becomes elongated in the direction of the flow-induced stress field (Tsai, 2012; Languin-Cattoën et al., 2021; Wang et al., 2022), potentially promoting clot formation. Elongated vWF fibers have been found inside the MemL (Steiger et al., 2019; Wagner et al., 2024). Still, there are no definitive results to date indicating that these high shear rates occur inside the MemL and that these are responsible for clotting. However, this could be a plausible mechanism for intra-device clot formation in ECMO despite the administration of anticoagulants. The detailed underlying mechanisms of these potentially life-threatening complications are not yet sufficiently understood and are the subject of intensive research (Murphy et al., 2015; Staessens et al., 2022). Increasing evidence points to the interplay between leukocytes, platelets, and the plasmatic coagulation cascade in the pathophysiology of clot formation (Massberg et al., 2010; Fuchs et al., 2010; Brill et al., 2012; Cerletti et al., 2012; Maugeri et al., 2014; Martinod and Wagner, 2014; Millar et al., 2016; Middleton et al., 2020).

The detection of adherent leukocytes (Lehle et al., 2008; 2016; Wilm et al., 2018) and platelet-leukocyte aggregates (PLA) (Wagner et al., 2024) on the hollow-fibers of MemLs as well as prothrombotic neutrophil extracellular traps (NETs) and their precursors *in-vitro* (Foltan et al., 2025) and in the blood of ECMO patients (Haus et al., 2024) provided further evidence for the involvement of immune cells in the formation of clots and deposits during ECMO therapy.

To date, these findings are limited to small studies, and larger, systematic investigations, particularly of the cellular deposits on the hollow-fibers of the MemL are lacking. Previous methods for analyzing these deposits in top-view microscopy images have primarily relied on manual procedures or rough estimations using basic image processing software (Wilm et al., 2018; Dropco et al., 2023; Wagner et al., 2024). Manual approaches are both time-consuming and highly subjective, as the heterogeneous size and morphology of deposits often impede clear classification. In particular, aggregated and overlapping structures are difficult to delineate even for trained observers and are strongly dependent on optimal contrast. Automated approaches published to date typically rely on defined intensity thresholding, making them highly sensitive to variations in image brightness and contrast. As a result, they often produce inconsistent and inaccurate segmentation outcomes. As these methods have only been applicable for well-spread, single-layered cells such as in blood smears, the differentiation of individual objects within nuclei clusters or overlapping regions in clinically used medical devices is frequently less reliable than with manual annotation.

The aim of this work was to develop an automated and reliable image processing tool tailored to evaluate the multilayered nuclei clusters found on hollow-fibers of ECMO MemLs that can operate within common lab environments with standard, non-specialized hardware. This would enable a high throughput image evaluation in a short period of time, with limited technical requirements. This tool is essential for increasing the reproducibility and reliability of future studies to provide standardized and quantitative results. Future studies involving the analysis of multiple MemLs are pending. Each MemL consists of 119 stacked fiber mats that are to be analyzed, resulting in a substantial amount of data.

To enable a high-throughput, standardized image analysis tool for this problem a deep learning (DL)-based algorithm was applied, since threshold-based approaches show significant deficiencies with the interpretation of multilayered nuclei clusters. Precise detection and segmentation of those structures may be the foundation of in-depth analysis of yet unknown clot mechanisms in ECMO. The tool was developed using currently available non-specialized equipment, with histological images taken top-view from the fiber mats and image acquisition parameters optimized to achieve the best image data possible. Ultimately, the developed software tool enables fast, standardized, and precise image analysis (less than 30 s, depending on the hardware) paving the way for the full-scale analysis of ECMO MemLs.

## 2 Methods

**FIGURE 1:**
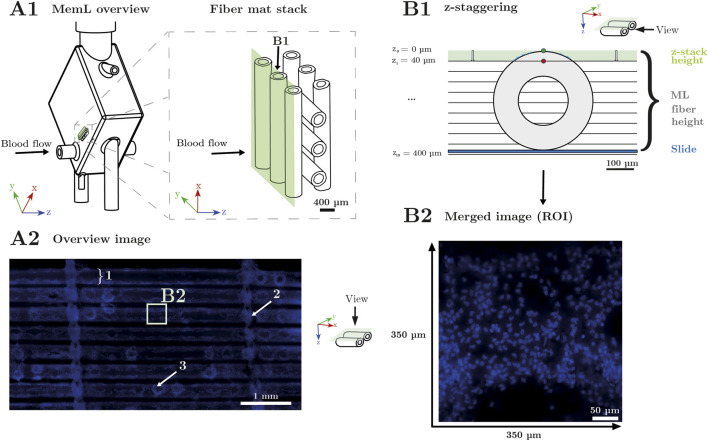
Image acquisition procedure. **(A1)** Schematic illustration of a Permanent Life Support MemL. Blood flow direction is indicated from left to right (arrow) and is further referred to as z-direction. When the MemL housing is opened, the MemL consists of stacked hollow-fiber mats. For visualization, an example of three fiber mats with three fibers each are displayed. **(A2)** Microscopic overview image of an excerpt of a single hollow-fiber mat in top-view acquired at 2.5× objective magnification. The sample consists of seven (1) hollow-fibers (horizontal). To form the fibers into a mat, they are connected by a woven (2) warp thread (vertical). Nuclear deposits are visualized by fluorescent staining using 4′,6-diamidino-2-phenylindole (DAPI, blue) and are mostly located on the fiber surface, particularly arranged circular around (3) crossing points with fibers of adjacent mats, and within the warp thread. This overview was used to select individual regions of interest (ROIs), which were centered on the surface of one hollow-fiber. **(B1)** Schematic illustration of a cross-section of one hollow-fiber. Selected ROIs were captured at a 40× objective magnification using z-staggering. Therefore, z_0_ was located just above the apex of the cylindric hollow-fiber and the microscope captured n = 150 consecutive images over a z-range of 40 μm; full z-stack height marked in green with z_0_ and z_1_ defining upper and lower limits of image acquisition; To ensure sharp focus throughout the fiber curvature these images were then processed to create a final composite image. **(B2)** Final composite image of a ROI with Extended Depth of Focus (EDOF) displaying sharply contoured nuclei.

The primary objective of the algorithm was to detect and localize nuclear deposits on fluorescence microscopy images of hollow-fibers taken from a clinically used, end of therapy VV ECMO Permanent Life Support (PLS)-MemL (Getinge, Rastatt, Germany; Figure 1). The MemL was collected from a 62 year old female patient after successful weaning with a total ECMO duration of 19 days. In the present case, clot formation was observed, as typically occurs in all patients. However, the extent of clotting remained below a severity level that would have necessitated a complete and clinically risky system replacement. Excessive clot burden markedly diminishes device performance, as it obstructs gas exchange and severely restricts blood flow, ultimately requiring full system exchange. Moreover, such extensive clotting would have precluded the generation of reliable ground truth (GT) labels: in heavily clotted regions, multiple densely packed and blurred nuclei layers become indistinguishable, making accurate manual annotation impossible. With moderate but still clearly visible clotting, however, GT generation remained feasible to a certain extent, enabling training the model to identify both isolated and aggregated nuclei.

**FIGURE 2:**
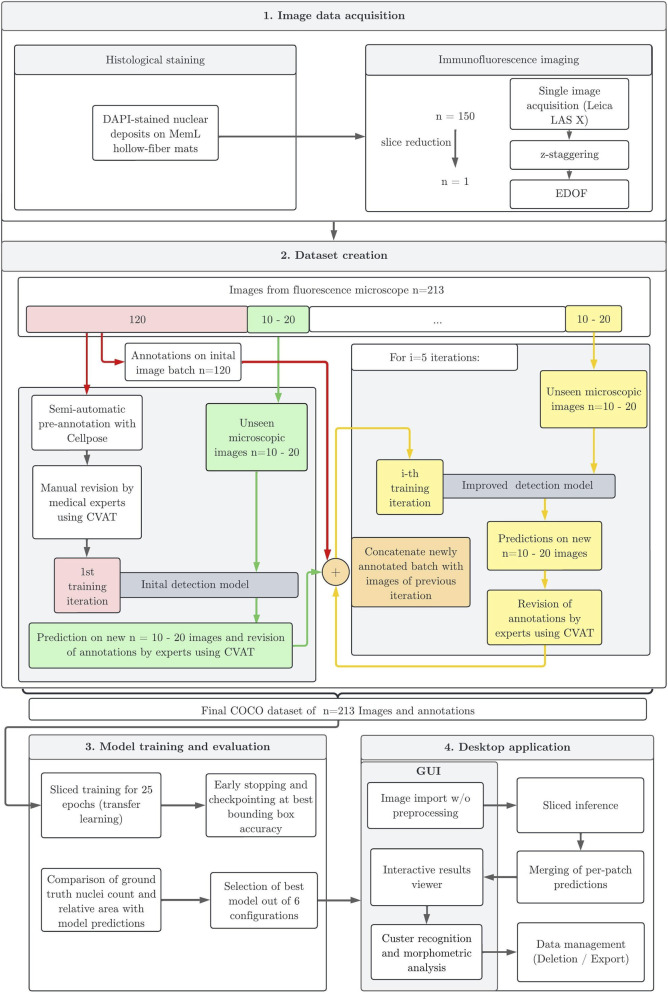
Illustration of the complete processing pipeline. Description from image acquisition over iterative labeling process and training, until the final model evaluation and implementation in a desktop application; DAPI: 4′,6-diamidino-2-phenylindole (DNA/nuclei staining), EDOF: Extended Depth of Focus, CVAT: Computer Vision Annotation Tool, COCO: Common Objects in Context.

To achieve this, a complete workflow was implemented, covering sample preparation, image acquisition, labeling, and the training and implementation of the DL model in desktop software. Figure 2 illustrates the entire processing pipeline, from generating the input image to producing the output from the desktop application. Data used for training as well as the source code of the desktop application are available under the GNU General Public License v3.0 on Zenodo as well as on GitHub (Pointner, 2026).

### 2.1 Sample preparation

The first step was to prepare the MemLs according to the protocol described by (Wagner et al., 2024). Ethical approval for the use of MemLs was obtained from the Ethics Committee of the University Regensburg (vote no. 20-2051-104). In brief, the entire MemL was thoroughly rinsed with isotonic saline immediately after termination of its clinical use to remove residual blood, followed by fixation with 4% paraformaldehyde to preserve cellular deposits on the fibers.

After fixation, the MemL was cut open to expose the 119 stacked fiber mats responsible for gas exchange and heat regulation during ECMO therapy. Each fiber mat was individually removed from the stack and sectioned into samples measuring 0.5 cm × 1.0 cm for subsequent staining and microscopic imaging. Only the opaque gas exchanging hollow-fibers were suitable for fluorescent staining as deposits from all sides of the fiber would be visible on transparent heat-exchanging hollow-fibers. All samples were consistently collected from a longitudinal region spanning from the device inlet to the device outlet. This ensured that the analyzed hollow-fiber sections represented the flow path along the primary direction of blood movement through the MemL. Importantly, no upper or lower peripheral regions (e.g., corner areas or edge zones of the fiber mat) were included in the sampling process. This approach was chosen to minimize variability arising from heterogeneous flow dynamics at the membrane periphery and to ensure that the analyzed samples reflected the physiologically most relevant flow trajectory.

To visualize cell nuclei and their remnants, the samples were mounted with the fluorescent DNA dye 4′,6-diamidino-2-phenylindole (DAPI; Fluoromount-G™ DAPI, 00-4959-52, Thermo Fisher, Waltham, MA, United States), as previously described (Wagner et al., 2024).

### 2.2 Image acquisition and data collection

The first step of the workflow depicted in Figure 2 involved the development of a new image acquisition procedure, for the generation of high-quality input data essential for high accuracy and robustness of the detection model. For this purpose, a dedicated fluorescence microscopy protocol was established using a Leica DMi8 inverted fluorescence microscope equipped with a LED 5 light source and a K5 grayscale camera (10x eyepiece). The microscope was fitted with a bandpass filter (420–450 nm) and a channel specific filter (420–460 nm) to enable selective excitation of the fluorophores as well as selective detection of their specific emission spectra. Image acquisition was performed using LAS X software (version 3.7.6.25997) (Leica microsystems GmbH, 2024).

During protocol development, special attention was given to minimizing imaging artifacts. Overexposure caused by excessive illumination intensities and long exposure times produced bright halos around the cell nuclei. These in turn reduced image quality by diminishing the contrast between nuclei and background and by blurring nuclear boundaries. Therefore, the acquisition parameters were optimized for the fluorescence channel to achieve the highest possible signal-to-noise ratio. DAPI-stained nuclei were captured at 40× objective magnification and were visualized in blue with an excitation wavelength of 390 nm (58% LED intensity and a 460-nm filter) and an exposure time of 250 ms. These optimized conditions minimized halo artifacts and produced high-contrast grayscale images suitable for reliable detection of DAPI stained nuclei as shown in Figure 1.

To ensure a representative and comparable evaluation across samples, a standardized image acquisition protocol was established. First, an overview image of the sectioned and stained hollow-fiber mat was acquired at 2.5× objective magnification (Figure 1A2). Areas containing warp threads, which connect the individual hollow-fibers to form the fiber mats, as well as crossing points between adjacent fiber mats, were excluded. This selection was made *a priori* based on methodological constraints. The analysis of nuclei structures at fiber contact points (CP) in the raw overview images was not possible with a high degree of reliability. The intense local brightness in these regions was attributable to the superimposition of multiple cell layers, resulting in the formation of highly condensed and irregular patterns. Consequently, nuclei boundaries either become indiscernible or indistinct, impeding reliable interpretation. Notably, even trained human observers encountered challenges in delineating individual nuclei in these regions with certainty. The incorporation of such domains into the manual annotation process would introduce significant subjective bias and result in a substantial decline in annotation quality. For this reason, only regions in which cell boundaries are clearly discernible and separable, i.e., between the CPs, were considered suitable for generating reliable GT. An area between the warp threads was selected, and a preview image at 10× objective magnification was obtained. For detailed analysis, ten regions of interest (ROIs) were acquired at 40× objective magnification per sample, deliberately omitting the hollow-fiber crossing points.

A z-stack acquisition was performed to obtain sharp images, even across the curved fiber surface (Figure 1B1). The z-range was defined between an upper limit (z_0_) just above the highest part of the fiber and a lower limit (z_1_) at 40 μm, corresponding to approximately one-sixth of the total fiber height. This range was automatically divided into 150 individual layers, with each layer focusing on a different depth plane of the fiber. The LAS X software then merged the layers into a single Extended Depth of Focus (EDOF) image, ensuring that cell nuclei at various depths were in focus (Figure 1B2). All images were saved in TIFF format with a resolution of 2,048 × 2,048 pixels and a bit depth of 16 bits. For subsequent annotation, the RAW files were converted to 8-bit images, as the labeling software (CVAT) did not support 16-bit formats.

### 2.3 Image labeling

After the image acquisition procedure, the gathered images were labeled to define the model’s prediction targets. To enable robust model training for the DL model and minimize manual labeling effort, a semi-automatic image annotation pipeline was developed, combining pretrained models with expert correction.

As shown in Figure 2, the initial segmentation masks in this work were generated using Cellpose v3.0.5 (Stringer et al., 2021). More specifically, the ‘Nuclei' model, which is based on a U-Net architecture, was used. U-Nets are convolutional neural networks (CNNs) widely used in biomedical image segmentation due to their efficiency with relatively small training datasets (Ronneberger et al., 2015). They are designed to work with a small amount of training data and are particularly effective for image segmentation. The Cellpose model provided initial segmentation masks for single nuclei in the images. However, it struggled with the correct detection of overlapping nuclei. The generated binary masks of the Cellpose model were converted to polygons and stored as JavaScript Object Notation (JSON) files in the COCO (Common Objects in Context) (Lin et al., 2014) format using a custom Python script. These annotations were formatted allowing for import and subsequent modification using Computer Vision Annotation Tool (CVAT) (CVAT.ai Corporation, 2024). In a second step, medical experts corrected missing or false annotations as well as mostly recognized but incorrectly pre-annotated overlapping nuclei clusters. Consistent and accurate labeling was ensured by manual quality control. The corrected annotations derived from the U-Net were then used to train a Mask R-CNN model. This model architecture was capable of both object detection and instance segmentation (He et al., 2017), providing bounding boxes and detailed segmentation masks. This dual output is advantageous because the bounding boxes can be used to support colocalization analysis.

In total 213 z-stacked 8-bit images (2,048 × 2,048 pixels) with a mean of 180 nuclei annotations were collected following the acquisition protocol. The dataset of 213 full-scale images was first split into training (80%) and validation (20%) subsets, with 170 full-scale images in train and 43 in validation. In the following, to reduce memory usage during training, image tiles of 400 × 400 pixels were created after the randomized train-test-split of the full-scale images with a fractional overlap of 20% (80 pixels in width and height), ensuring sufficient contextual information at the tile edges using the Slicing Aided Hyper Inference (SAHI) package (Akyon et al., 2022). Sliced windowing resulted in 8,330 training and 2,107 validation tiles, with 65,974 and 17,464 annotations, respectively (mean nuclei count: 7.92 for training and 8.29 for validation per tile). To accelerate the generation of the dataset, an iterative labeling approach was applied, illustrated in Figure 2. First, Cellpose-generated masks for 120 images were manually reviewed and corrected in CVAT. This dataset was used to train an initial “weak” model to generate higher-quality predictions, especially for complex cell conglomerates that are difficult to segment with conventional U-Net models. In each subsequent iteration, batches of 10–20 new unseen images were analyzed using the most recent model. The resulting predictions were converted into instance masks, reimported into CVAT, and manually revised by medical experts. The corrected annotations were then merged with the existing dataset to create an expanded and higher-quality training set. To further enhance segmentation performance, especially for clustered nuclei, an improved Mask R-CNN model was trained in each cycle. For every iteration, training was performed from scratch using an updated 80:20 training–validation split, discarding previous checkpoints. This iterative loop was repeated five times, progressively increasing the dataset size (e.g., 120 → 140 → … 213 images). This strategy substantially reduced labeling time compared to fully manual annotation, while improving segmentation quality.

Due to the limited amount of nuclei annotation data, transfer learning and finetuning weights was implemented within the detectron2 framework (Wu et al., 2019). Pretrained COCO ImageNet weights were fine-tuned for the specific task, enabling robust model adaptation despite the small dataset size. Although weak model checkpoints had to be discarded after each iteration, the iterative workflow substantially reduced manual annotation effort. As segmentation accuracy improved with each cycle, manual correction became progressively less necessary, and intermediate model predictions already surpassed the quality of the initial Cellpose pre-annotations. The final detection model was trained on image tiles derived from 170 full-scale images and evaluated on 43 validation images (n = 213 total).

### 2.4 Model training

**FIGURE 3:**
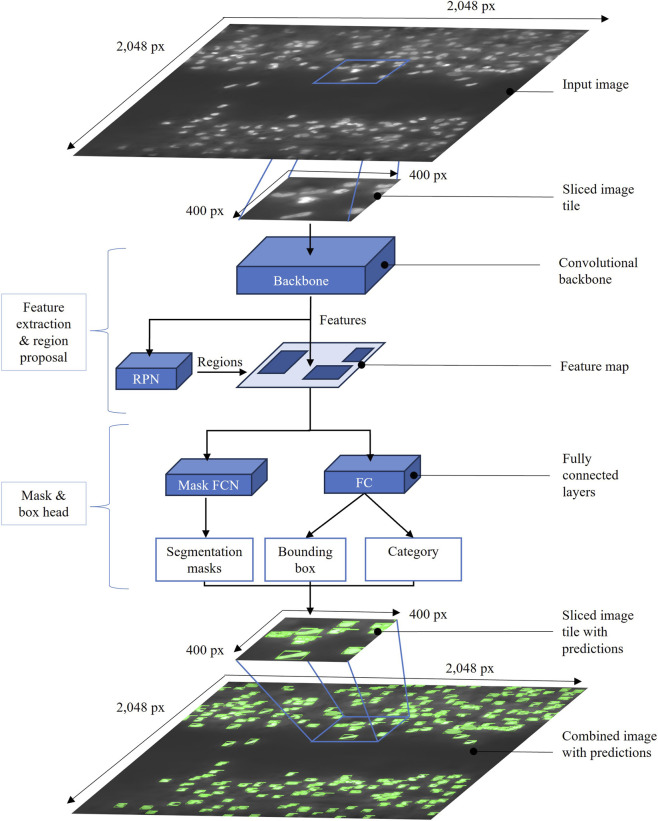
Overview of the Mask R-CNN architecture used for nuclei detection. To reduce memory usage, input images were segmented into 400 × 400-pixel tiles with 20% fractional overlap for nuclei detection. The backbone served as a feature extractor. A Region Proposal Network (RPN) suggests candidate object regions. The Mask R-CNN model includes two task-specific heads: a Fully Convolutional Network (FCN) for predicting pixel-level masks and a network with fully connected layers for regressing bounding boxes and classifying each predicted instance. Bounding boxes and segmentation masks visualized in light green. Six different configurations of the model were tested in total, with the DC5 variant performing best. Finally, the per-tile predictions from the image tiles were merged into a combined image to recreate the original image. Nuclei predictions located on multiple tiles were merged into one.

The selected detection model was capable of both object detection and instance segmentation, enabling precise localization of individual nuclei and distinction of overlapping structures. The overall architecture and processing workflow, from input image to final prediction, is illustrated in Figure 3. Model training was conducted using the detectron2 framework (Wu et al., 2019), which provides flexible configuration options and efficient implementation for Mask R-CNN training. The model uses a CNN as its backbone for hierarchical feature extraction. The extracted features are then processed by two task-specific branches: one for generating bounding boxes and classification outputs (in this case, ‘nucleus’ as the single class) and another for predicting pixel-wise segmentation masks corresponding to each detected instance.

To evaluate the influence of different backbone architectures on instance segmentation performance, several Mask R-CNN configurations were tested. Specifically, ResNet-50 (R_50) and ResNet-101 (R_101) backbones were used, the numbers 50 and 101 refer to the network depth. Originally introduced for the ImageNet classification task, ResNet architectures remain among the most widely used backbones in computer vision due to their strong feature extraction capabilities (He et al., 2016). ResNet architectures are widely used in computer vision. This network is composed of residual blocks, consisting of three sequential convolutional layers with skip connections that sum the feature maps elementwise, facilitating gradient flow during training.

Within the Mask R-CNN framework, various network heads were explored for bounding box generation and mask prediction to assess the efficacy of nuclei detection performance of each network backbone. The following architecture configurations were utilized.

- C4: Based on the initial implementation of Faster R-CNN (Ren et al., 2015), this configuration uses the output of the fourth convolutional stage (conv4) of the ResNet backbone as the feature extractor. The term R_50_C4 describes this setup when using the ResNet-50 architecture.
- DC5: The dilated convolution (DC) variant of the standard convolution algorithm (Yu and Koltun, 2016) expands the receptive field by inserting bypassed gaps between kernel elements. In this configuration, dilation is applied to the fifth convolutional stage (conv5) of the ResNet. The setup R_50_DC5 refers to the combination of ResNet-50 with this dilation strategy.
- FPN: The Feature Pyramid Network (FPN) (Lin et al., 2017) is a feature extraction module that generates multi-scale feature maps from a single-scale image using a bidirectional pathway. The bottom-up pathway operates as a standard CNN, generating feature maps at various scales, with each stage’s final residual block output serving as feature activations for ResNet. The top-down pathway upsamples higher level feature maps and merges them with lower-level activations from the bottom-up pathway through lateral connections. FPN configurations are labeled with a “_FPN” suffix.

Training was conducted with a base learning rate of 0.002 and a batch size of one image tile per iteration. A total of 25 epochs were completed on an NVIDIA A100 Graphics Processing Unit (GPU). Model evaluation on both training and validation was performed after every epoch (every 8,329 iterations). Model accuracy was assessed using the COCO evaluation Application Programming Interface (API), which evaluates performance based on the Intersection over Union (IoU) metric across multiple thresholds (IoU from 0.5 to 0.95). The average precision (AP) at a single IoU threshold of 0.50 (AP^IoU=.50^) served as the primary performance metric for both bounding boxes and segmentation masks. Automatic checkpointing was applied according to the best bounding box (bbox) accuracy (bbox_val) as precise nucleus localization was of primary interest. Detailed spatial information of individual nuclei instances allows for later colocalization analyses and the integration of multiple color channels, e.g., for additional fluorescence staining. Final training resulted in six model configurations: R_50_C4, R_50_DC5, R_50_FPN, R_101_C4, R_101_DC5 and R_101_FPN. To increase the variety of input data, data augmentation was conducted. However, only random horizontal flipping was ultimately applied, as additional methods (e.g., color jittering or random cropping) did not improve segmentation performance.

### 2.5 Model validation

Statistical analysis was performed using the Mann–Whitney U test to compare the results of the Mask R-CNN models with different backbones against the expert annotations (GT) as well as against Cellpose and StarDist (Schmidt et al., 2018). For Cellpose, the ‘Nuclei’ model type was used with the ‘diameter’ evaluation parameter set to its default value of 30. With StarDist (v0.9.2), the pretrained ‘2D_versatile_fluo’ model was applied with the default settings without specifying any additional input parameters.

A p-value of less than 0.05 was considered statistically significant. Relative area values refer to the proportion of either the GT annotation area or the area of predicted cell segmentation mask with respect to the total image area. Results of the statistical comparison can be found in Figure 5.

### 2.6 Model application as part of a desktop program

A sliced inference approach was introduced to reduce the GPU memory usage during model inference and to enable the GUI software to run efficiently on lower-performance GPUs (e.g., NVIDIA Quadro P620). Input images were divided into 400 × 400-pixel tiles, processed individually and subsequently reconstructed by aggregating per-tile predictions (Figure 3). This tiling strategy also allowed for parallelized inference and multiple trial creation within the desktop application. To enhance detection precision, bounding boxes with confidence scores below 98% were discarded using non-maximum suppression (NMS), thereby favoring fewer but more reliable detections. During reconstruction, non-maximum merging (NMM) was applied with an intersection over smaller area (IoS) threshold of 0.5. This procedure merged overlapping predictions from adjacent tiles based on the smaller of the intersecting areas, reducing duplicate detections while preserving spatial accuracy.

**FIGURE 4:**
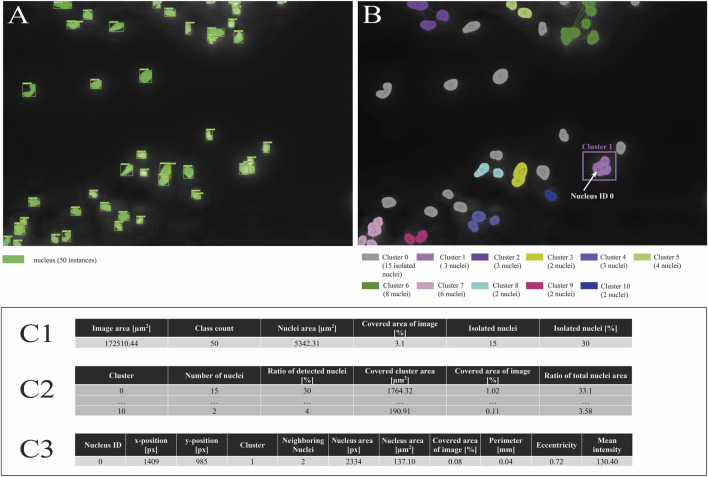
Postprocessing of model predictions. **(A)** Exemplary model predictions as displayed in the desktop software, bounding boxes and segmentation masks visualized in light green. **(B)** Detection of isolated (gray) and clustered (colored) nuclei. **(C1–C3)** Samples from the image-specific results of nuclei morphometric analysis of nuclei. **(C1)** Summary per image examined. **(C2)** Detailed information on recognized clusters, including the total and relative area covered by detected nuclei. **(C3)** Sample of extensive information per recognized nucleus instance, covering individual nucleus location on the image, number of neighboring cells, nuclei perimeter, and eccentricity.

After segmentation and detection of the nuclei, various post-processing steps were performed allowing for detecting nuclei clusters, isolated cells as well as describing the morphology of individual nuclei. Firstly, a graph-based clustering algorithm identified spatially or geometrically associated nuclei (Figure 4B). For each pair of nuclei, a check was performed to determine whether they either have a relevant mask overlap (>2%) or are within an adaptive distance threshold, which is adjusted dynamically, based on nuclei radii and local density. If at least one condition was met, nuclei were considered connected, and an edge was inserted between the corresponding nodes in the associated graph. Clusters were identified as connected components within this graph, with isolated cells receiving cluster ID 0 and related groups being numbered sequentially. For each cluster, aggregated parameters such as the absolute number of nuclei and the total image area covered by the cluster, as well as the ratio of the cluster’s nuclei count to all detected nuclei and their occupied area in relation to the full-scale image, were calculated to characterize the nuclei distribution and structure (Figure 4C2). Secondly, various morphological and spatial characteristics were calculated for each individual nucleus (Figure 4C3). Geometric descriptors included area, position on the image, perimeter and nucleus eccentricity. Eccentricity is derived from an ellipse fit to the segmentation mask, with values closer to zero denoting circular nuclei, while values approaching one indicate elongated shape. Moreover, mean color intensity within each mask is extracted to capture fluorescence intensity characteristics. Spatial features such as local nuclei density (the number of neighboring cells in the vicinity) as well as the distance to the nearest neighbor provide further quantitative context for cellular distribution.

In summary, the developed program proved capable of detecting and counting fluorescence-stained cell nuclei as well as their remnants and performing subsequent analyses such as nuclei morphology characterization. To enable the evaluation of spatial relationships between individual nuclei within the deposits, the selected model architecture supports both object detection and instance segmentation. Additionally, a graphical user interface (GUI) was developed to facilitate interaction with the DL model and to visualize the algorithm’s output (Supplementary Figure S1). Post-processing delivers outputs at both macro (image-level) and micro (nucleus-level) scales, enabling quantitative assessment of nuclear features such as size, eccentricity, and brightness. Clustering further examines spatial relationships, for instance by estimating neighboring cells to identify isolated nuclei (Figure 4). These functionalities are unique to our nuclei detection approach and are not available in other tools such as Cellpose and StarDist.

## 3 Results

**FIGURE 5:**
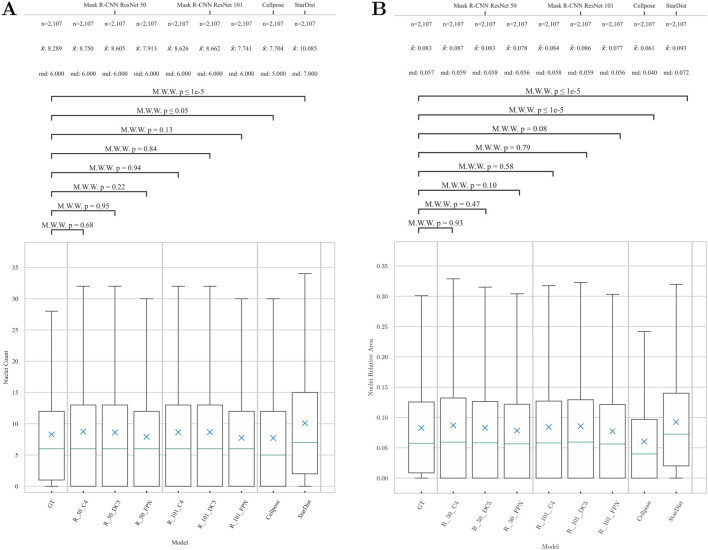
Statistical comparison of model configurations. Results of six Mask R-CNN model configurations with GT in terms of **(A)** nuclei count and **(B)** relative area per tile (n = 2,107). Blue cross: mean ( $\bar{x}$ ); Blue horizontal line: median (md); Box indicates values between first and third quartile. Whisker bars: 1.5 times interquartile distance. Values of the mean and median given above. To compare the models to the GT, Mann-Whitney-U Test (MWU) (p-values) was used. On average, image tiles contained $\bar{x}$ = 8.29 nuclei, occupying a relative tile area of 8.3%. Of the six configurations that were selected, none demonstrated statistical significance in relation to GT. The Mask R-CNN with Resnet 101 DC5 backbone was selected as the preferred model, representing an optimal compromise between accurate nuclei count (p = 0.84) and adequate detected nuclei area (p = 0.79). Cellpose demonstrated an acceptable degree (p ≤ 0.05) of accuracy in cell nucleus counting; however, the segmented cell nucleus area exhibited a significant deviation from the GT (p ≤ 1e-5). StarDist predicted a significantly higher number of instances than all the other models (p ≤ 1e-5). Furthermore, the area of nuclei predicted by StarDist is greater than that predicted by GT (p ≤ 1e-5).

**FIGURE 6:**
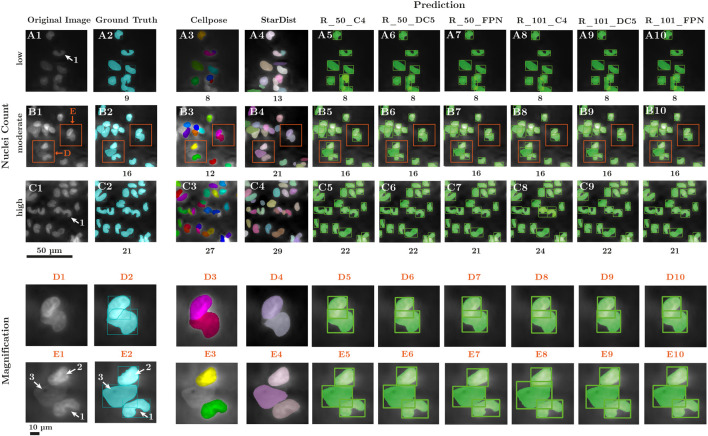
Comparison of nuclei segmentation across model configurations. **(A–C)** Representative examples of three 400 × 400-pixel image tiles and **(D, E)** magnified views of cell clusters as indicated in tile **(B)**. **(A1-E1)** Original gray scale images showing nuclei on a fiber of a membrane lung; nuclei visualized with fluorescent DNA staining using DAPI; of particular interest were overlapping nuclei **(D)** and nuclei with irregular form (segmented, u-shaped nucleus; marker 1) or different staining intensity: nuclei with high DNA-density provided good contrast (marker 2) while low DNA-density resulted in nuclei outlines difficult to discern (marker 3). **(A2-E2)** Nuclear outlines and associated boxes provided by medical experts, presenting the GT for model training **(A-E 3–10)** Predictions from multiple model configurations; Nuclei count of GT and all model predictions presented under each tile **(A3-E3)** Cellpose, an existing nuclei detection model trained for single layered, well spread nuclei. Yet, in samples of clinically used materials, Cellpose presented inaccuracies in correctly detecting overlapping nuclei and nuclei with u-shaped form or low DNA-density **(A4-E4)** Prediction of the StarDist model; like Cellpose, incorrectly detected u-shaped nuclei. Overall, more instances were predicted in relation to GT. In comparison, Mask R-CNN results using the ResNet 50 backbone **(A5-E5)** R_50_C4 **(A6-E6)** R_50_DC5 **(A7-E7)** R_50_FPN, and ResNet 101 backbone **(A8-E8)** R_101_C4 **(A9-E9)** R_101_DC5 **(A10-E10)** R_101_FPN. All models outlined nuclei very similarly to the GT. Magnified views highlight improved detection of nuclei with low DNA-density and segmentation of overlapping or irregular nuclei by all Mask R-CNN variants compared to Cellpose and StarDist.

A total of six Mask R-CNN model variants were trained on the 8,330 tiles of 170 full-scale images. Each model produced bounding boxes identifying individual nuclei within the images, along with their corresponding segmentation masks. Post-processing was then applied to generate additional outputs, including the total count and area of nuclei, as well as detailed information for each nucleus (location, size, perimeter, eccentricity) and their colocalization within cell clusters (Figure 4). To enable the use of detected nuclei in other applications, each individual nucleus mask could be exported both as a JPEG image and in COCO format. For a validation set comprising n = 2,107 image tiles derived from n = 43 full-scale validation images, the performance of six Mask R-CNN model configurations was evaluated with respect to nuclei count and the relative area of predicted nuclei compared with GT annotations (Figure 5). A visual comparison of nuclei segmentation across model configurations with respect to GT, and other state-of-the-art tools such as Cellpose and StarDist is provided in Figure 6.

### 3.1 Quantitative evaluation

Overall, all six Mask R-CNN models demonstrated strong performance in detecting nuclei on clinically used specimens. As shown in Table 1, none of the trained models exhibited a significant deviation from the GT in terms of nuclei count or relative nuclear area per tile (0.08 ≤ p ≤ 0.95). Among the models, R_50_DC5 provided the most accurate nuclei count predictions, whereas the ranking based on relative nuclear area differed, with R_50_C4 performing best in this category. The bounding box accuracy (bbox_val, AP^IoU=.50^) identified R_50_C4 as the top-performing model, closely followed by R_101_C4, R_50_DC5 and R_101_DC5. Segmentation accuracy (segm_val, AP^IoU=.50^) similarly showed that R_50_C4 and R_101_C4 achieved the highest performance, followed by R_50_DC5 and R_101_DC5. Mask R-CNN models demonstrated superior performance compared to Cellpose’s U-Net, primarily due to improved correspondence with GT nuclei segmentation areas. Although Cellpose achieved acceptable nuclei count accuracy (p = 0.05), particularly for nuclei with high DNA density that provided strong contrast (Figure 6, marker 2), its overall segmentation quality was markedly inferior. Specifically, Cellpose frequently fragmented irregular U-shaped nuclei into two separate masks (Figure 6A1–A3, marker 1), whereas all Mask R-CNN variants consistently segmented these nuclei as single instances (Figure 6A5–A10, E5-E10). Furthermore, nuclei with low DNA density, appearing dim (Figure 6, marker 3), were often not captured by Cellpose (Figure 6E3), while Mask R-CNN models detected them reliably (Figure 6E5–E10). These limitations—fragmentation of irregular nuclei, omission of overlapping regions, and failure to detect dim nuclei—resulted in a significantly reduced relative segmentation area compared to GT (Figure 5, p ≤ 1e-5). In comparison, the Mask R-CNN variants prediction demonstrated a high degree of correspondence with the GT mask area (0.08 ≤ p ≤ 0.95). While StarDist like Cellpose delivered acceptable segmentation results of nuclei with high DNA density that provided strong contrast (Figure 6, marker 2), it predicts comparably more instances on the image tiles, than Cellpose and the Mask R-CNN variants, ultimately increasing the predicted nuclei count (Figure 5A, p ≤ 1e-5) and nuclei area (Figure 5B, p ≤ 1e-5). This is mostly due to the prediction of nuclei in areas not labeled as “nuclei” (Figure 6A4–C4) when compared to GT (Figure 6A2–C2).

**TABLE 1:** Performance of Mask-RCNN model in comparison to Cellpose and StarDist.

Trained mask r-cnn model configurations	Statistical comparison		Detection/Segmentation accuracy	
	Nuclei count (p-value) vs. GT	Nuclei area (p-value) vs. GT	bbox_val (AP^IoU=.50^)	segm_val (AP^IoU=.50^)
R_50_C4	0.68	0.93	94.51	94.22
R_50_DC5	0.95	0.47	93.87	93.99
R_50_FPN	0.22	0.10	93.37	93.63
R_101_C4	0.94	0.58	93.92	94.22
R_101_DC5	0.84	0.79	93.81	93.97
R_101_FPN	0.13	0.08	93.31	93.56

References nuclei model	Statistical comparison			
	Nuclei count (p-value) vs. GT	Nuclei area (p-value) vs. GT		
Cellpose	0.05	1e-5	n.a	n.a
StarDist	1e-5	1e-5	n.a	n.a

### 3.2 Qualitative evaluation

Upon comparison of the quantitative results of all models, their qualitative output for nuclear outline was compared to the expert opinion and the already available software Cellpose and StarDist. Three randomly selected tiles in Figure 6 illustrate representative input data for the models as processed during training. This allows small-scale visual inspection and comparison of actual model predictions. In fluorescence microscopy input images (Figure 6A1–E1), nuclei varied substantially in shape, size, local density, and brightness mostly due to fluctuations in DNA-density and therefore different fluorescence signal strength. Manual detection of the nuclear outlines (GT, Figure 6A2–B2) was not limited by this fluctuation of signal strength. Cellpose (Figure 6A3–E3) was able to detect isolated nuclei, yet split curved or u-shaped nuclei into multiple instances, leading to an increased nuclei count. Additionally, nuclei with low DNA-density were neglected, reducing the nuclei area and count (Figure 6A3). Additionally, StarDist appears to demonstrate deficiencies in its capacity to detect and segment u-shaped nuclei (Figures 6A4–C4). Further, it predicted areas in the images as nuclei, not present in GT, ultimately increasing predicted nuclei count and area (Figure 6A4–C4).

In contrast, the different Mask R-CNN configurations (Figures 6A–E 5–10) demonstrated superior instance separation and contour accuracy, particularly in low-signal regions. Magnified views in the bottom two rows (Figures 6D,E) highlight improved segmentation of overlapping or irregular nuclei by all Mask R-CNN variants compared with Cellpose and StarDist. All models produced similar nuclei counts. However, subtle variations in segmentation quality were observed, suggesting superior nuclei delineation performance of certain models.

### 3.3 Full-scale reconstruction

**FIGURE 7:**
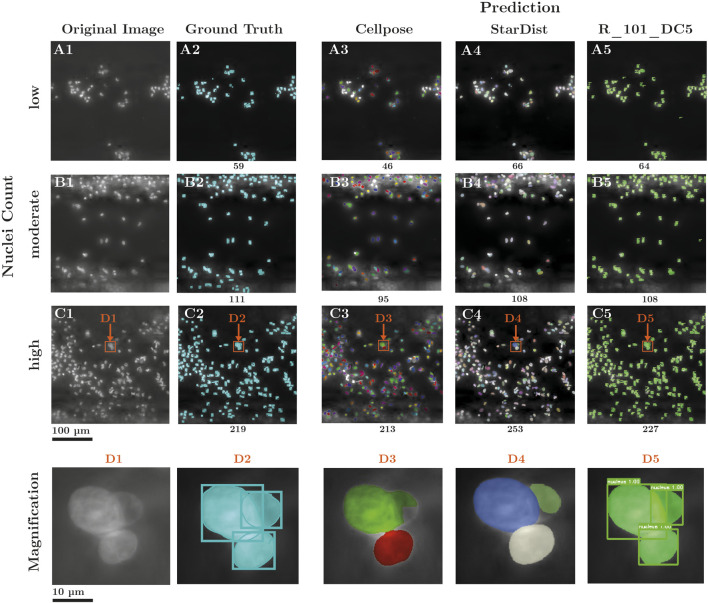
Full-scale evaluation of the Cellpose and R_101_DC5 model prediction. **(A1-C1)** Full-scale input images (2,048 × 2,048 pixels) with low, medium and high nuclei count **(A2-C2)** GT annotations made by clinical experts. Predictions of the **(A3-C3)** Cellpose and **(A4-C4)** StarDist model **(A5-C5)** predictions of the selected R_101_DC5 model as yielded after post-processing by the desktop program. Predictions were derived from a tile basis and reconstructed to full image dimensions. **(D1-D5)** Magnification of selected ROI, with nuclei overlapping. **(D3)** Cellpose detected only two instances, with the mask of the upper one overestimated in terms of area, while the underlying one was neglected. **(D4)** StarDist detected three instances, however, the nuclei intersection was not considered. **(D5)** In contrast, R_101_DC5 correctly detected three instances and their overlap.

Per-tile predictions were recombined into full-scale images using the described reconstruction pipeline (Figure 3). The results, exemplarily shown in Figure 7 generated with the R_101_DC5 model, demonstrated consistent alignment between predicted outlines and GT annotations, including post-processing steps such as NMS and NMM. An exploratory analysis across three images with regions with varying local nuclei densities (Figure 7) revealed that the selected model tended to slightly overestimate nuclei counts in sparse and densely populated areas and underestimate them in medium-density regions. Although these examples represent only a subset of the data, they illustrate the model’s consistent performance across heterogeneous input microscopy images. The Cellpose model demonstrates a high degree of accuracy in detecting isolated cells; however, it exhibits challenges in detecting nuclei with relatively strong DAPI-signal (Figure 6B3, top region) or blurred nuclei (Figure 7C3,D3). StarDist predicted more instances on images with low nuclei density (Figure 7A4) compared to Cellpose (Figure 7A3) and R_101_DC (Figure 7A5). On images with high nuclei density, it over-segments, with the most predicted instances in comparison (Figure 7C4). Like Cellpose, overlaps between nuclei were not captured correctly (Figure 7D4). In contrast, the selected Mask R-CNN model with a DC5 backbone demonstrated a superior ability to capture nuclei overlaps (Figure 7D5).

### 3.4 Applicability of the model to other acquisition cases

**FIGURE 8:**
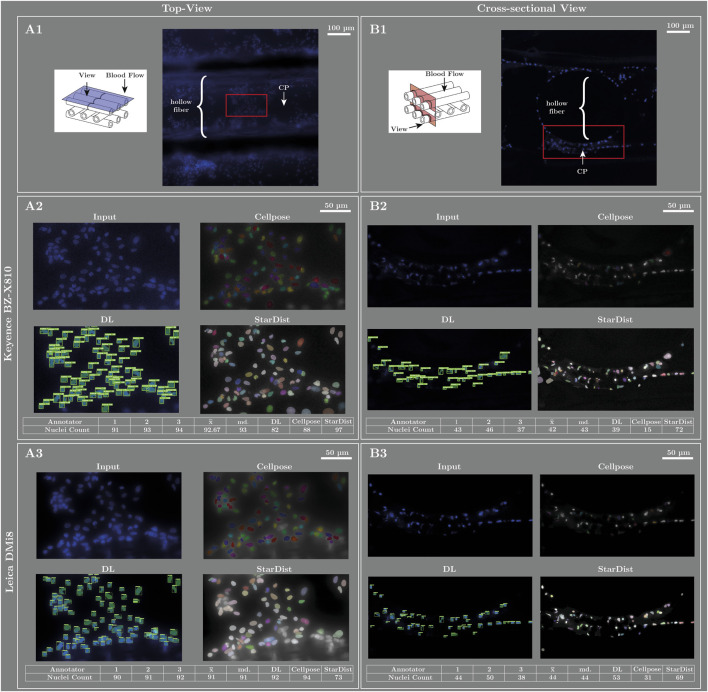
Evaluation of model generalization capability across two fluorescence microscope systems and acquisition settings. Two almost identical sample regions were imaged using a Leica DMi8 and a Keyence BZ-X810, both equipped with a 10x eyepiece at 40× objective magnification. The left column shows the top-view acquisition, while the right column shows the cross-sectional perspective obtained from a polymer-embedded MemL sample (prepared according to (Kranz et al., 2026). In each view, overview images were captured with 10× objective magnification **(A1, B1)**, the ROI is displayed in red, with hollow fiber structures and CP annotated for orientation, alongside a pictogram indicating the viewing direction on the hollow-fiber mats. The ROI was analyzed using our DL software, Cellpose, and StarDist **(A2, A3, B2, B3)**. Acquisition settings differed between microscopes: Leica images were recorded at 250 ms exposure time and 2,048 × 2,048 pixels, while Keyence images used 5 ms (top view) and 6.67 ms (cross-section) exposure times at 960 × 720 pixels. For evaluation, the blue RGB image was used as model input. Manual nuclei count from three independent medical experts, who did not participate in GT generation, are shown below the output images alongside the counts produced by the software tools.

To evaluate the model’s capacity for generalization across various microscope systems and acquisition settings, the same sample region was imaged with two fluorescence microscopes: the Leica DMi8 and the Keyence BZ-X810. The Keyence microscope is currently in use for scientific research at the University Hospital Regensburg. To eliminate potential bias, laboratory personnel unaffiliated with the authors conducted the image capturing. Both systems were equipped with a 10x eyepiece. The analysis comprised two complementary perspectives. First, the hollow-fiber mat was imaged from top-view at 40× objective magnification. Second, the cell layer was examined from an orthogonal acquisition direction. To accomplish this, a polymer-embedded MemL sample was used, following the embedding and sample preparation procedure described by (Kranz et al., 2026) This polymeric embedding process allowed for the first time to visualize the cross-section of clot burden on ECMO MemL hollow-fiber mats. The resulting images are presented in Figure 8. The left column shows the top-view acquisition, and the right column shows the cross-sectional perspective obtained through polymeric embedding. Figure 8A1 and B1 show 10x overview images with the ROI highlighted in red. We subsequently analyzed this ROI using our software tool, as well as with the open-source segmentation models Cellpose and StarDist (Figure 8 A2,A3,B2,B3). For orientation purposes, both the hollow-fiber and the contact point (CP) are annotated in each view. Moreover, a small pictogram indicates the viewing direction on the fiber mats.

The acquisition parameters differed between the two microscope systems. The Leica microscope captured the top-view and cross-section images with an exposure time of 250 ms (acquisition parameters according to (Kranz et al., 2026). In contrast, the Keyence top-view image (Figure 8A2) was recorded with an exposure time of 5 ms (shutter speed: 1/200 s), and the cross-section image was recorded with an exposure time of 6.67 ms (shutter speed: 1/150 s). Image resolution also varied substantially between the two microscope systems: the Leica microscope produced 2,048 × 2,048-pixel images, whereas the Keyence system generated images at 960 × 720 pixels. For this evaluation, we directly used the blue channel of the RGB images as input, which differs from the grayscale inputs used during model training and for the statistical comparison. In addition to the automated analyses performed by the software tools, three independent medical experts were asked to manually count the nuclei instances to provide reference values. These experts were not involved in the GT generation and therefore contributed an independent bias distinct from any bias potentially introduced during the original labeling process. The counts obtained from the three medical experts, as well as those generated by the software tools, are reported below the corresponding output images. “DL” denotes our DL–based software tool.

## 4 Discussion

The present work introduces an automated methodology for high-quality fluorescence microscopic imaging and nuclei detection. Particular focus was on the visualization of nuclei on hollow-fibers from clinically used ECMO MemLs. By integrating a customized image acquisition pipeline, an iterative semi-automated labeling strategy, and DL–based image segmentation, this work establishes an efficient workflow for analyzing complex nuclear deposits within MemLs (Figure 2). Unlike *in-vitro* studies or blood smear samples, where cells are evenly spread across a flat surface, nuclear deposits on clinically used MemL hollow-fibers presented multiple difficulties: First, cells were attached to a three-dimensional cylindric hollow-fiber, and second, they were not spread out evenly but arranged in cell clusters. This resulted in overlapping nuclei and different staining intensities between highly crowded areas and isolated nuclei. Previously published cell detection software like Cellpose and StarDist struggled to provide satisfactory nuclei detection in these specific specimen. Therefore, previous studies investigating cellular and thrombotic deposits on MemL hollow-fibers have largely relied on highly time-consuming manual or threshold-based analyses (Wilm et al., 2018; Dropco et al., 2023; Wagner et al., 2024). These methods were limited by subjectivity, error proneness, and low scalability, making systematic analyses of larger datasets infeasible. Considering findings highlighting the role of leukocytes and in particular their interplay with platelets and plasmatic coagulation in ECMO-associated coagulation disorders (Lubnow et al., 2014; Murphy et al., 2015; Staessens et al., 2022; Haus et al., 2024), the presented workflow provides a technically robust foundation for future large-scale studies investigating MemL deposits.

An iterative labeling process significantly reduced manual annotation time and improved dataset quality through expert refinement. Depending on the cell occurrence, manually labeling a full-scale image took between two and four hours, even after pre-annotation, which illustrates the considerable amount of resources and time required. Despite its limited size, we have ensured that the dataset captures a level of diversity relevant to the task. The dataset includes both areas with significantly lower cell density (Figure 6A1) and highly condensed nuclei areas (Figure 6C1). This distribution provides heterogeneous examples of nuclei arrangements. The nuclear instances exhibit a variety of shapes, including circular (Figure 6A1–B1) and u-shaped (Figure 6A1, marker 1). Furthermore, the instances have different brightness values ranging from dark-appearing instances with low DAPI signal to bright instances with stronger DAPI signal. The images include cell arrangements with varying degrees of overlap and spatial organization, as well as varying signal intensities, ensuring that the model is exposed to different morphological and contextual conditions. The resulting dataset was used to train six Mask R-CNN instance segmentation model configurations (Figure 3).

Further, the developed GUI enables intuitive model interaction, automated nuclei detection, and morphometric analysis (Supplementary Figure S1). Post-processing generates output at both macro (image-level) and micro (nucleus-level) scales, allowing quantitative evaluation of nuclear features such as size, eccentricity, and brightness. Clustering further analyzes spatial relationships, for example, by estimating neighboring cells to identify isolated nuclei (Figure 4). This data provides a foundation for advanced investigations, including PLA and NETosis detection. NETotic cells often exhibit spatial isolation, enlarged morphology, and reduced brightness (Brinkmann and Zychlinsky, 2007); however, additional fluorescence markers are required for robust classification as previously described (Haus et al., 2024). The software outputs positional, morphological, and intensity data for each nucleus, supporting high-throughput analysis and enabling preliminary identification of potential NETotic cells or PLAs. This highlights the need for subsequent in-depth studies and refined classification strategies.

Transfer learning enabled the utilization of a modestly sized dataset, yet it maintained high segmentation accuracy for a great spectrum of different sized objects, as evidenced in the COCO challenge, but also for the more intricate task of segmenting small nuclei in fluorescence images. Moreover, Mask R-CNN showed superior ability to handle heterogeneity in nuclear size, shape, and fluorescence intensity compared to Cellpose and StarDist (Figures 6, 7). The presented models performed particularly well in two critical regions: High- and low-signal areas. In regions with high cell density and therefore high fluorescence signal, such as overlapping cells or larger cell clusters, the models provided accurate outlining of adjacent and curved nuclei that were often misclassified by traditional threshold-based methods. Further, the localization of nuclei is detected and allowed an allocation with other nuclei in clusters and can be used for co-localization with other blood components. This makes the models attractive for large-scale investigation of cellular attachment on *in-vivo* blood-stream devices, where nuclei are naturally distributed. On the other hand, nuclei with low DNA-density and therefore low fluorescence signal were detected successfully by R-CNN models unlike by previously published software. Unique to our approach is the possibility to extract and describe nuclear morphology (individual nuclei location, perimeter, eccentricity, color intensity). The occurrence of enlarged nuclei with reduced DNA-density has been of particular interest in the investigation of NETosis (Brinkmann and Zychlinsky, 2007). Enlarged nuclei have been observed in MemLs (Wagner et al., 2024; Foltan et al., 2025), yet they have been manually registered and without further objective morphologic assessment.

Among the six model configurations, the R_50_DC5 model achieved the highest correspondence with GT nuclei counts, while R_50_C4 performed best in terms of detected nuclei area. R_101_DC5 provided the best balance between nuclei count and relative area predictions (final validation accuracy (AP^IoU=.50^): bbox_val 93.81% and segm_val 93.97%). The R_101_DC5 backbone, employing dilated convolutions, preserved higher spatial resolution during feature extraction and avoided excessive down-sampling. Together with the integration of five ResNet convolutional layers, this design facilitates the generation of richer feature representations. Consequently, the DC5 configuration enabled the Mask R-CNN model to detect and segment even densely packed or overlapping nuclei, as frequently encountered on clinically used MemL hollow-fibers. Cellpose’s U-Net struggled to accurately segment irregularly shaped nuclei and detect nuclei with low DNA density. Fragmentation of u-shaped nuclei into multiple segmentation masks and omission of dim nuclei were common, particularly in regions with overlapping structures. These limitations resulted in significantly reduced segmentation area compared to GT (p ≤ 1e-5). In contrast, the selected Mask R-CNN R_101_DC5 variant consistently preserved complex nuclear morphology and detected both dim and irregular shaped nuclei, achieving superior correspondence with GT segmentation area (p = 0.79). Minor discrepancies between reconstructed full-scale images and GT annotations (Figure 7) in terms of nuclei counts can be attributed to the tiling strategy used during training and inference. The tiling is required during training as full-scale images could not be forwarded to the Mask R-CNN due to memory constraints. The slicing approach also allowed the detection software to be run on lower-end hardware. Post-processing threshold-based steps such as NMS and NMM effectively minimized redundant detections, although occasional merging artifacts at tile borders may have slightly influenced cell counts. Further optimization may improve the merging of sliced predictions. In contrast, Cellpose does not apply slicing of input images or prediction merging, so it avoids reconstruction to the original image dimensions. While Cellpose’s total nucleus count is closer to the GT, this metric alone does not make the Cellpose model more accurate than the Mask R-CNN approach. Cellpose generates incorrect segmentation masks more frequently than Mask R-CNN, particularly in dense, crowded nuclei clusters (Figure 7D3). This leads to inaccurate area measurements and reduced computed area, as well as inaccurate per-cell segmentation, which is required for subsequent nuclear morphological examination and classification. However, Cellpose often neglects nuclei instances, especially blurry and bright ones (Figures 7A3–C3), which reduces the total nuclei count. StarDist, on the contrary, predicted comparably more nuclei on both tile (Figures 6A4–C4) and full-scale level (Figure 7A4–C4), leading to an increased nuclei count and relative area (Figure 5). While these detection errors may partially compensate for each other in aggregate metrics, they result in unreliable spatial and morphological information per instance. In contrast, our pipeline provides accurate area calculations and preserves structural integrity by delivering more precise per-cell mask segmentation, particularly for overlapping nuclei. This ensures higher-quality data for downstream analysis.

For detailed observation of specimen oriented perpendicular to the main flow direction, like the vicinity of CPs between two fiber mats, we further advanced a newly developed embedding procedure that enables three-dimensional, high-resolution analysis of complex clotting structures, which has recently been published (Kranz et al., 2026). Here also multi-layer nuclei clusters occur that were processable with our tool. However, for observing the clot burden in flow direction, we rely on top-view images used for GT generation, subsequent model training and development of the processing software. Using both the embedding procedure and the processing software allows the evaluation of clots in MemL in both perspectives, allowing for in-depth high-throughput clot analysis.

Beyond ECMO-specific applications, the developed workflow demonstrates how DL techniques can overcome the limitations of manual or threshold-based segmentation in biomedical microscopy. The presented software particularly enables the objective and fast detection of nuclear deposits on clinically used blood-stream devices. The model was trained and validated using images acquired from a single microscope system under optimized and standardized acquisition conditions. CPs between fiber mats were excluded during GT generation because manual delineation of individual nuclei in these regions was not feasible. To further evaluate the model’s generalization capability, we performed an additional assessment using two distinct samples imaged with two different fluorescence microscope systems. For these analyses, reference nuclei counts were provided by three independent medical experts who were not involved in the DL model training or labeling process. Due to the differing acquisition characteristics of the two microscopes, obtaining perfectly identical images of the same sample was not methodologically feasible. However, capturing nearly equivalent regions allowed us to assess how well the model identifies nuclei under substantially different imaging conditions.

For the top-view sample captured with the Keyence microscope (Figure 8A2), the DL model produced fewer instance masks than the expert counts, whereas Cellpose and StarDist yielded counts more closely aligned with the expert evaluations. In relative terms, the our trained DL model deviated from the median of the expert nuclei counts by 11.8% (DL: 82, md.: 93) for the Keyence top-view images (Figure 8A2). Our model intentionally filtered out very blurry nuclei that experts still included in their subjective manual counts; by contrast, StarDist frequently detected these poorly defined structures. The reduced count from our model is likely attributable to the exclusion of blurry instances during labeling, the lower spatial resolution of the Keyence images (960 × 720 pixels vs. 2,048 × 2,048 pixels from the Leica system), and the substantially shorter exposure time used during the Keyence acquisition (5 ms vs. 250 ms). Notably, the counts by the experts themselves vary from person to person across samples, indicating the strong subjective bias inherent in manual nuclei counting.

In the cross-sectional sample, our DL model accurately detected even highly irregular nuclei and produced a count closest to the expert evaluations. In contrast, Cellpose missed many nuclei (relative 65,1%; Cellpose: 15, md.: 43), whereas StarDist tended to over-segment the images (relative 67,4%, StarDist: 72, md.: 43; Figure 8B2,B3). Overall, our model demonstrated robust nuclei detection on images acquired under markedly different imaging parameters. In relative terms, for the Keyence cross-sectional images (Figure 8B2) our DL model deviated by 9.3% (DL: 39, md.: 43) from the median of the expert nuclei counts. In general, it provided a standardized, non-threshold-based analysis that consistently disregarded very blurry instances while still correctly detecting overlapping nuclei. Furthermore, it proved capable of identifying irregularly shaped nuclei, including those distorted through slicing or sample preparation (Figure 8B2,B3). The combination of automated threshold-independent detection, instance segmentation, spatial analysis and morphological details allows for comprehensive characterization of complex cellular structures in large datasets, offering significant potential for translational and experimental research.

## 5 Conclusion

This work presents a workflow for the automated analysis of nuclear deposits on fluorescence microscopy images of ECMO MemL hollow-fibers. This enables nuclei detection, quantification and morphological assessment in naturally distributed deposits on medical devices. The framework integrates a customized image acquisition pipeline, iterative labeling as well as the use of a DL-based segmentation model. A semi-automated, iterative labeling strategy was performed to generate a comprehensive dataset, which served as the foundation for training multiple configurations of a Mask R-CNN instance segmentation model.

In total, six different Mask R-CNN variants were developed and compared to results of medical experts and the already available Cellpose and StarDist software. Mask R-CNNs variants outperformed both the Cellpose and StarDist model for our application. In particular, the DC5 configuration of the Mask R-CNN architecture, with ResNet 101 as feature extractor, proved highly effective for nuclei detection and segmentation in challenging fluorescence microscopy images. Its ability to preserve spatial resolution through dilated convolutions and extract fine morphological details enables accurate feature identification of nuclei even of irregular, u-shape, reduced brightness or overlapping instances in densely packed nuclei clusters. These were cases where Cellpose’s and StarDist’s accuracy was lacking. The developed tool, complemented by a user-friendly GUI, facilitates automated detection, quantification, and morphological assessment of nuclei. The algorithm’s capacity to retain positional information for each detected nucleus facilitates not only quantitative but also spatial analyses of nuclei aggregation. Additional morphological analysis allows for subsequent in-depth nuclei classification. The developed software tool facilitates rapid, consistent, and accurate image analysis (with a duration of less than 30 s, depending on the hardware) and paves the way for comprehensive analysis of ECMO MemLs.

Future work may focus on expanding the framework to multi-channel fluorescence imaging for cell-type differentiation, implementing overlap-aware labeling for improved segmentation in crowded regions, and developing 3D reconstructions for spatial modeling of nuclei distribution. We performed assessment of the model’s capability to generalize across varying capturing setups and acquisition settings, where it proved to detect nuclei also on images with differing exposure time and image resolution. Beyond ECMO applications, the approach can be readily adapted to other biomedical imaging tasks, such as histological tissue sections, or cell culture studies, where reliable detection and characterization of nucleated cells are essential.

## Data Availability

The raw data supporting the conclusions of this article have been made available by the authors without restriction under the GNU General Public License v3.0 on Zenodo and GitHub (Pointner, 2026).
