# Supplementary figures and images for: Automated deep learning based detection of cellular deposits on clinically used ECMO membrane lungs
Source: Front Bioinform. 2026 Apr 1;6:1771574. doi: 10.3389/fbinf.2026.1771574 (PMC13079351; doi:10.3389/fbinf.2026.1771574)

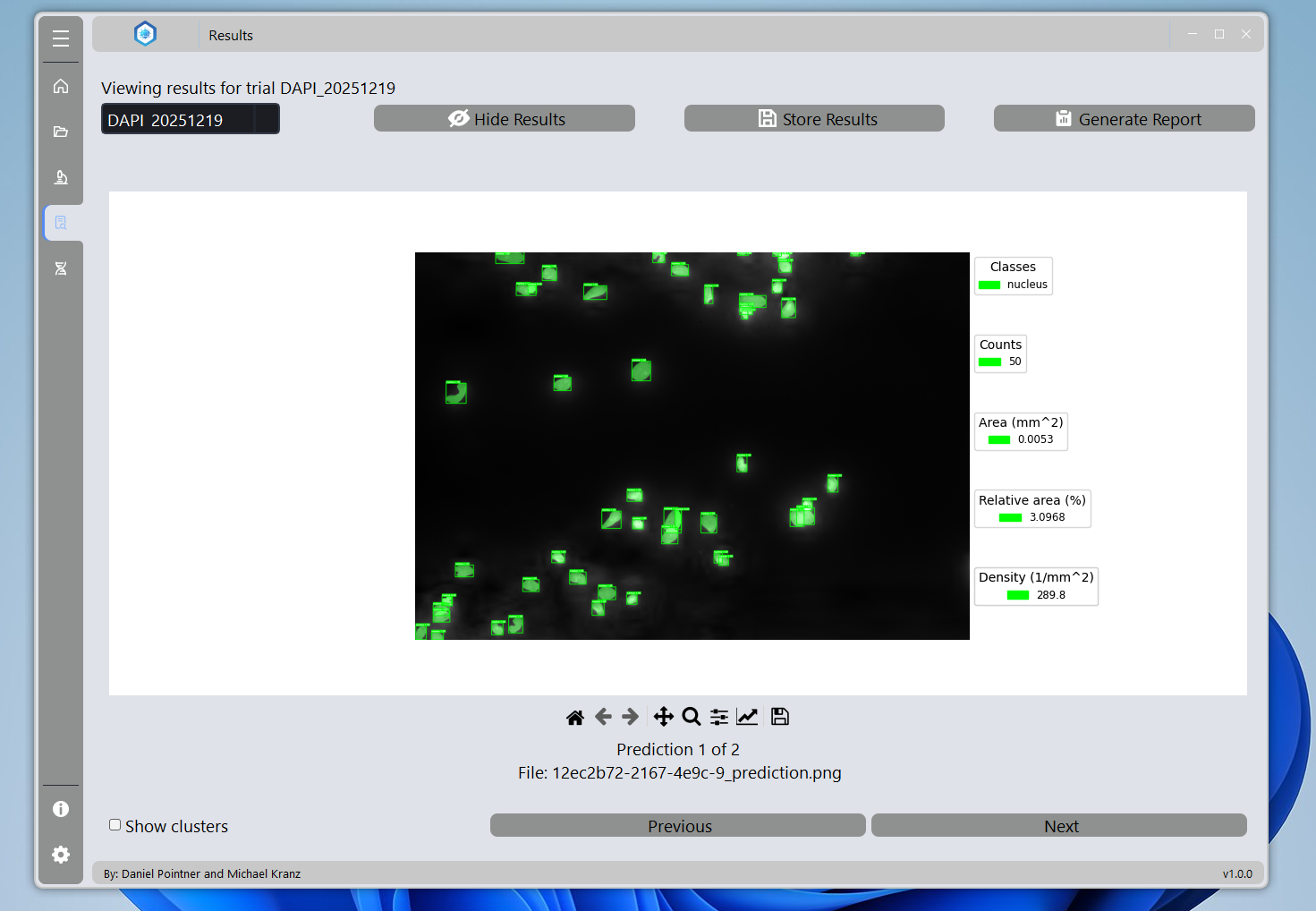

Supplement: Supplementary file 1 [file Image1.png]
